# Supplementary material for: Leptodactylus latrans Amphibian Skin Secretions as a Novel Source for the Isolation of Antibacterial Peptides
Source: Molecules. 2018 Nov 11;23(11):2943. doi: 10.3390/molecules23112943 (PMC6278411; doi:10.3390/molecules23112943)
Supplement: Supplementary file 1 [file molecules-23-02943-s001.pdf]

Supporting Information

Leptodactylus latrans Amphibian Skin Secretions as a novel source for the isolation of Antibacterial Peptides (Anura: Leptodactylidae)

Alvaro Siano[†≠]*, Humpola Veronica*[†≠]*; Spinelli, Roque*[†]*, Eliandre de Oliveira*[§]*, Fernando Albericio*[⊥,||,∇]*,, Simonetta, Arturo*[‡]*; Rafael Lajmanovich* [O ≠] *and Georgina G. Tonarelli*[†*]*.*

[†]Departamento de Química Orgánica, Facultad de Bioquímica y Cs. Biológicas (FBCB), Universidad Nacional del Litoral (UNL). Ciudad Universitaria, (3000) Santa Fe, Argentina.

[O]Cátedra de Ecotoxicología, Escuela Superior de Sanidad. FBCB, U.N.L. Ciudad Universitaria, (3000) Santa Fe, Argentina.

[§]Proteomics Platform, Barcelona Science Park, Baldiri Reixac 10, 08028-Barcelona, Spain.

[⊥]Institute for Research in Biomedicine and CIBER-BBN, Baldiri Reixac 10, 08028-Barcelona, Spain.

[||]Department of Organic Chemistry, University of Barcelona, 08028-Barcelona, Spain.

[∇]School of Chemistry and Physics, University of KwaZulu-Natal, 4000-Durban, South Africa.

[‡]Cátedras de Microbiología y Biotecnología, Departamento de Ingeniería en Alimentos, Facultad de Ingeniería Química, U.N.L. Santiago del Estero 2829, (3000) Santa Fe, Argentina.

[≠]Consejo Nacional de Investigaciones Científicas y Técnicas (CONICET), Argentina.

Table of Contents

Figures	Content	Page
**S1**	Analysis of the MS-MS spectra of Seq.2. Theoretical MW: 1577.8	3
**S2**	Analysis of the MS-MS spectra of Seq.1. Theoretical MW: 1298.5	4
**S3**	Chromatographic profile of the three synthetic peptides	5

Table	Content	Page
**S1**	**Table S1.** Alignment of the ion with m / z: 1298.72 with the amino acid sequences of the six ocellatins reported from *L. latrans*	6

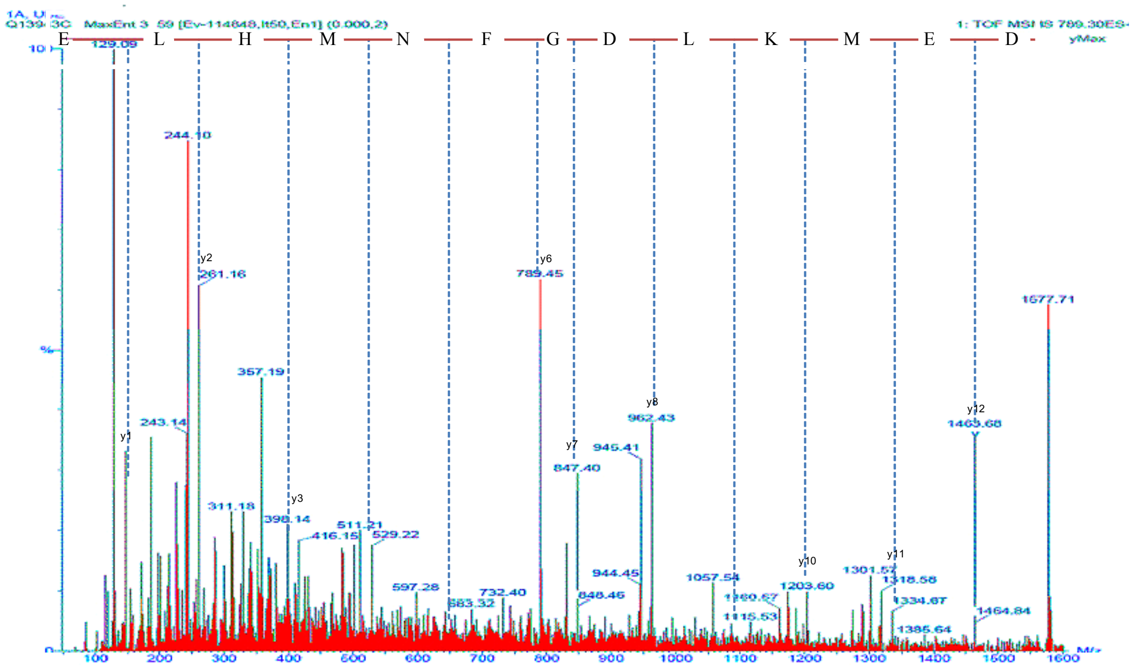

Figure S1. Analysis of the MS-MS spectra of Seq.2. Theoretical MW: 1577.8

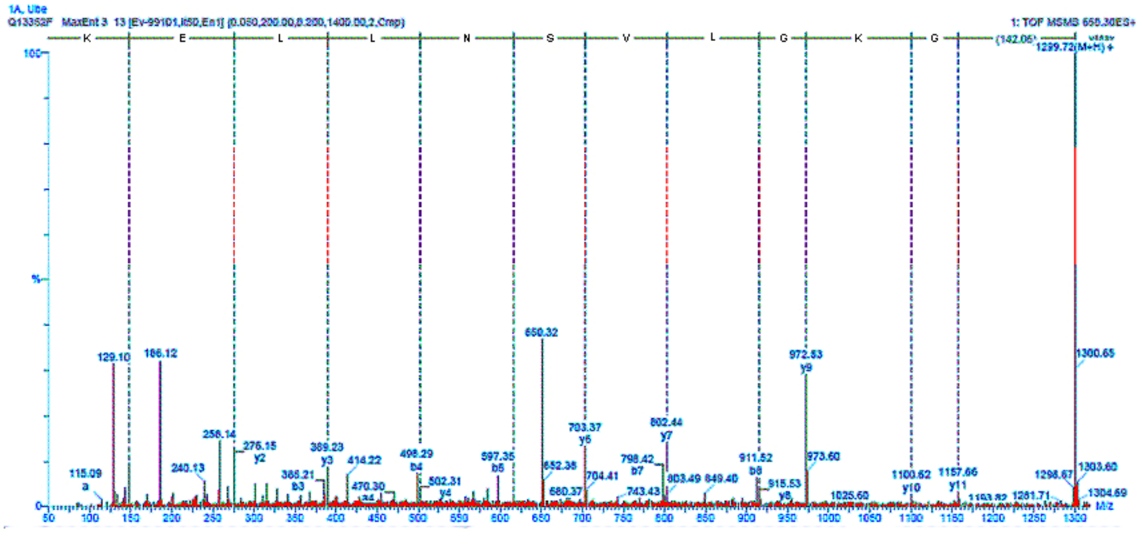

Figure S2. Analysis of the MS-MS spectra of Seq.1. Theoretical MW: 1298.5

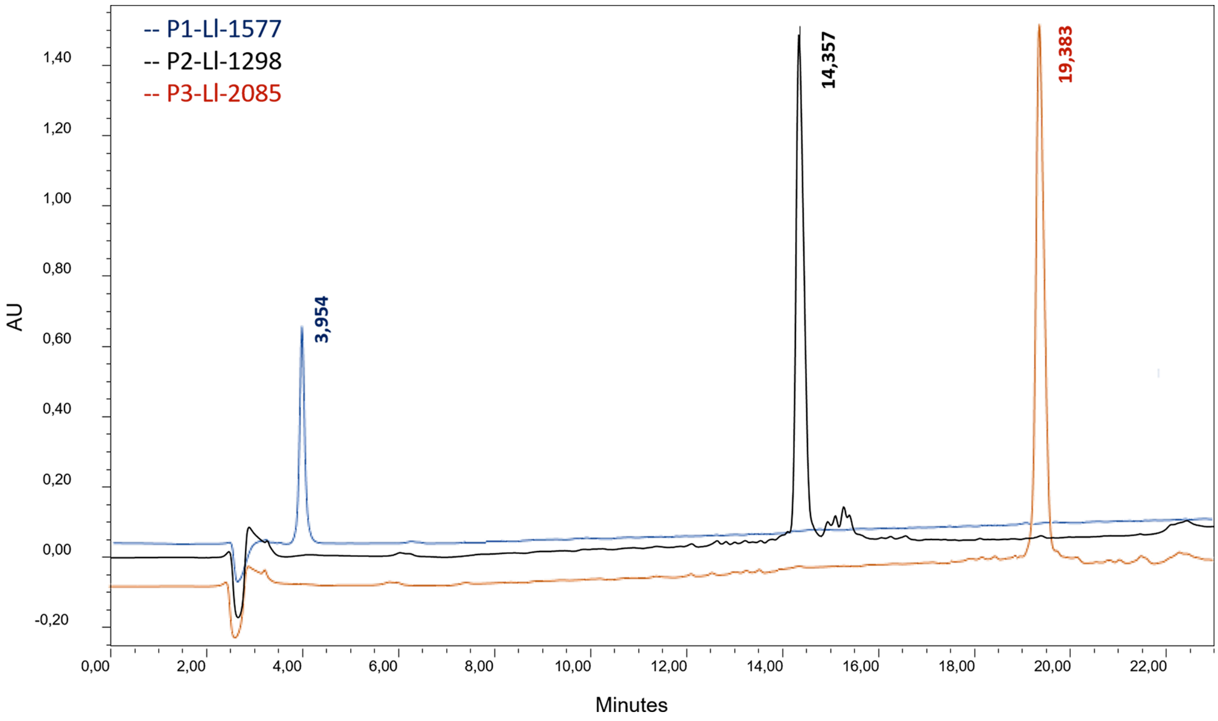

Figure S3. Chromatographic profile of the three synthetic peptides.Column: C_{18} Jupiter Proteo Phenomenex. Gradient: 5-70% ACN: H_2O. Detection: 220 nm.

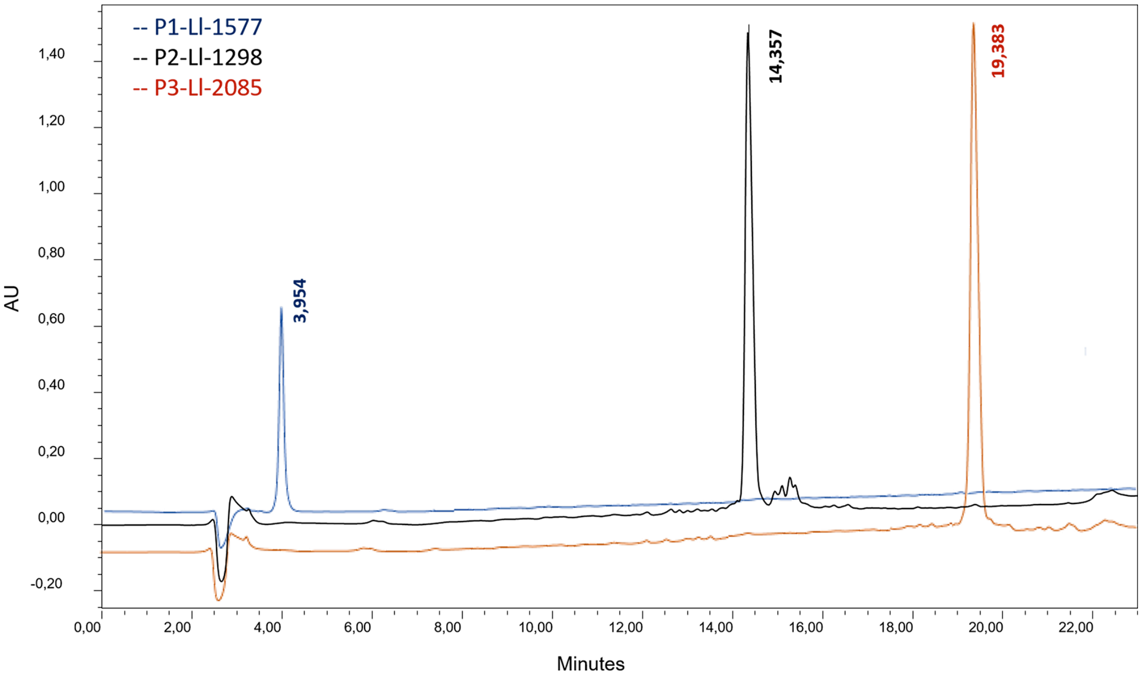

Table S1. Alignment of the ion with m / z: 1298.72 with the amino acid sequences of the six ocellatins reported from *L. latrans*

Peptide	Amino acid Sequence																									Nr
**Ocellatin-1**	**G**	V	V	**D**	I	L	K	G	**A**	**G**	**K**	D	**L**	L	A	H	**L**	V	G	K	I	S	E	K	V	25
**Ocellatin-2**	**G**	V	L	**D**	I	F	K	D	**A**	A	**K**	Q	**I**	L	A	H	A	A	E	Q	I	-	-	-	-	21
**Ocellatin-3**	**G**	V	L	**D**	I	L	K	N	**A**	A	**K**	N	**I**	L	A	H	A	A	E	Q	I	-	-	-	-	21
**Ocellatin-4**	**G**	L	L	**D**	F	V	T	G	V	**G**	**K**	D	**I**	F	A	Q	**L**	I	K	Q	I	-	-	-	-	21
**Ocellatin-5**	**G**	L	L	**D**	F	L	K	**A**	**A**	**G**	**K**	**G**	**L**	**V**	**T**	**N**	**L**	-	-	-	-	-	-	-	-	17
**Ocellatin-6**	A	V	L	**D**	F	I	K	**A**	**A**	**G**	**K**	**G**	**L**	**V**	**T**	**N**	**I**	M	**E**	**K**	V	G	-	-	-	22
**Ion 1299 .72 (S1)**	-	-	-	-	-	-	-	**A**	**A**	**G**	**K**	**G**	**L**	**V**	**S**	**N**	**L**	L	**E**	**K**	-	-	-	-	-	13

Alignment using the UniProt database (Universal Protein Resource 2011, http://www.uniprot.org/). Nr: Number of amino acid residues.
